# Malignant Right Atrial Mass With Inferior Vena Cava Extension Presenting as Budd-Chiari Syndrome: A Multimodality Imaging Case Report

**DOI:** 10.7759/cureus.96345

**Published:** 2025-11-07

**Authors:** Mohamed Ztati, Wissame Dahmane, Walid Ait Moha, Mustapha EL Hattaoui

**Affiliations:** 1 Cardiology, Hospital ARRAZI CHU Mohammed VI UCA FMPM, Marrakesh, MAR

**Keywords:** budd–chiari syndrome, cardiac angiosarcoma, cardiac magnetic resonance, computed tomography, echocardiography, inferior vena cava extension, multimodality imaging, right atrial tumor, tumor thrombus, young adult

## Abstract

A 29-year-old man with no significant past medical history presented with progressive abdominal pain, ascites, and New York Heart Association (NYHA) class III exertional dyspnea. Clinical evaluation suggested portal hypertension, and imaging confirmed extensive inferior vena cava and hepatic vein thrombosis consistent with Budd-Chiari syndrome. Transthoracic echocardiography revealed a large right atrial mass extending from the inferior vena cava, raising suspicion for a malignant cardiac tumor. Cardiac magnetic resonance imaging (MRI) and computed tomography (CT) further characterized the lesion with features of malignancy, without evidence of extra-cardiac primary disease. Coronary angiography unexpectedly demonstrated chronic right coronary artery occlusion with well-developed collateral circulation. Laboratory investigations excluded common thrombophilic and autoimmune causes of venous thrombosis. The patient was initiated on anticoagulation and supportive therapy; however, rapid clinical deterioration precluded surgical resection.

This case highlights a rare presentation of malignant right atrial tumor manifesting as Budd-Chiari syndrome in a young patient. It emphasizes the diagnostic value of multimodality imaging and the need for early recognition and multidisciplinary decision-making in managing such highly lethal conditions.

## Introduction

Cardiac masses comprise a heterogeneous group of lesions, including neoplasms, thrombi, and pseudotumors, each with distinct prognostic and therapeutic implications [1].

Primary cardiac tumors are exceedingly rare, with a reported prevalence of 0.0017%-0.028% in autopsy and surgical series [1,2]. Among these, malignant forms account for roughly one quarter, most commonly sarcomas arising from the right atrium [3]. However, secondary involvement or direct tumor extension from adjacent structures may produce overlapping imaging features, making the distinction between primary and secondary lesions particularly challenging in the absence of histopathological confirmation [4].

Clinical manifestations are often nonspecific and may reflect systemic venous congestion, pulmonary embolism, or obstructive phenomena rather than cardiac symptoms [5,6]. In rare cases, patients can initially present with features of Budd-Chiari syndrome - a manifestation typically associated with hepatic venous outflow obstruction [7,8].

Here, we report the case of a young patient presenting with Budd-Chiari syndrome secondary to a right atrial mass extending into the inferior vena cava (IVC). This case highlights the diagnostic challenge of distinguishing tumor thrombus from bland thrombus and illustrates the crucial role of multimodality imaging in patient management.

## Case presentation

In May 2025, a 29-year-old man with no known cardiac, hepatic, or renal disease was admitted for progressive abdominal pain, distension, and dyspnea over seven months, complicated two months prior by altered consciousness and severe dyspnea (NYHA IV). He had no personal or family history of thrombophilia or autoimmune disease, except for a father with documented gastric malignancy.

On examination, he was conscious but asthenic, normotensive (BP 110/70 mmHg), tachycardic (HR 96 bpm), and maintaining SpO₂ 98% on room air. Notable findings included a distended abdomen with flank dullness, prominent collateral veins along the flanks, and right upper quadrant tenderness, findings consistent with venous congestion. No peripheral edema, cyanosis, or jugular venous distension was observed.

Basic laboratory tests revealed leukocytosis, mild renal impairment, hypoalbuminemia, and elevated inflammatory markers, consistent with systemic congestion. 

A comprehensive immunologic and thrombophilia workup (including antinuclear antibody (ANA), anti-double-stranded DNA (anti-dsDNA), antineutrophil cytoplasmic antibody (ANCA), antiphospholipid antibodies, factor V Leiden, prothrombin gene mutation, proteins C/S, antithrombin III, and JAK2 V617F) was negative, excluding autoimmune or inherited hypercoagulable states.

Vital signs and detailed laboratory results at admission and follow-up are summarized in Table 1.

**Table 1:** Vital signs and comprehensive laboratory results at admission and follow-up, including hematologic, renal, hepatic, lipid, immunologic, thrombophilia, and tumor marker panels, as well as ascitic fluid analysis. Reference ranges are shown for comparison. ​CRP, C-reactive protein; ALAT (ALT), alanine aminotransferase; ASAT (AST), aspartate aminotransferase; GGT, gamma-glutamyl transferase; LDH, lactate dehydrogenase; NT-proBNP, N-terminal pro b-type natriuretic peptide; AFP, alpha-fetoprotein; CEA, carcinoembryonic antigen; CA, cancer antigen (CA 15-3, CA 19-9, CA 125, CA 72-4); PSA, prostate-specific antigen; β-hCG, beta-human chorionic gonadotropin; ANA, antinuclear antibodies; Anti-DNA, anti-double-stranded DNA antibodies; ANCA, anti-neutrophil cytoplasmic antibodies; Anti-SM, anti-smooth muscle antibodies; Anti-LKM1, anti-liver kidney microsomal antibodies type 1; Anti-β2GP1, anti-beta-2 glycoprotein I antibodies; EPP, electrophoresis of plasma proteins, Prothrombin G20210A, prothrombin gene mutation (guanine-to-adenine substitution at position 20210); JAK2 V617F, Janus kinase 2 gene mutation (valine-to-phenylalanine substitution at codon 617).

Category	Parameter	Result	Units	Reference range
Vitals	Blood pressure	110/70	mmHg	90-120 / 60-80
	Heart rate	96	bpm	60-100
	SpO₂ (room air)	98	%	≥95
	Temperature	36.6	°C	36-37.5
	Glasgow Score	15/15		15
Hemogram	White blood cells (WBC)	17,970	/µL	4,000-10,000
	Hemoglobin	13.6	g/dL	13-17 (male)
	Platelets	168,000	/µL	150,000-400,000
Coagulation	Prothrombin time	28.2	%	70%-100%
Inflammatory	CRP	40	mg/L	<5
Renal	Urea	0.21	g/L	0.15-0.45
	Creatinine	14.63	mg/L	6-13
	Albumin	22	g/L	35-50
Hepatic	ASAT	146 (×5N)	U/L	<35
	ALAT	28	U/L	<45
	Alkaline phosphatase	265 (×1.5N)	U/L	<120
	GGT	102(x2N)	U/L	<50
	Total bilirubin	52	µmol/L	<17
	LDH	756 (×3N)	U/L	140-280
Immunology	ANA (IFI)	<80 (negative)		Negative <80
	Anti-DNA	<10 (normal)	IU/mL	Negative <10 UI/mL
	ANCA (P/C)	Negative		Negative
	Rheumatoid factor	<8.0 (negative)	IU/mL	<14
	Anticardiolipin abs	<20 (negative)	GPL	Positive >40
	Anti-B2GP1 abs	12 (negative)	GPL	Positive >20
	Lupus anticoagulant	NR < 1.2 (negative)		Positive: NormalizedRatio>1.2
	Anti-SM abs	1:30 (negative)		Positive > 1:80
	Anti-LKM1 abs	<1:10 (negative)		Positive>1:40
Complement	C3; C4	1.1 (normal); 0.3 (normal)	g/L	0.9-1.8; 0.1-0.4
Thrombophilia	Protein C activity	100%	%	70-140
	Protein S activity (free)	90%	%	60-150
	Antithrombin III activity	110%	%	80-120
	Factor V Leiden mutation	Not detected		
	Prothrombin G20210A mutation	Not detected		
	JAK2 V617F mutation	Not detected		
	Homocystein	10	µmol/L	5-15
Tumor markers	Alpha-fetoprotein	3.82 (normal)	ng/mL	<10
	BHCG	1 (negative)	mUI/mL	Normal < 5
	CEA	2.84 (normal)	ng/mL	<5
	CA 15-3	29,4 (normal)	U/mL	<30
	CA 125	942.8 (elevated)	U/mL	<35
	CA 19-9	102 (elevated)	U/mL	<37
	PSA total	1.42 (normal)	ng/mL	<4
	PSA free	0.2 (normal)	ng/mL	>0.25 ratio
Other	NT-proBNP	100	pg/mL	<125
	EPP	Beta-gamma block		-
Ascitic fluid	Appearance	Yellow citrin		-
	Proteins	6.8	g/L	Transudate <25
	LDH	109	U/L	<250
	WBC	158	/µL	<250
	Lymphocytes	81%	%	-

**Figure 1:**
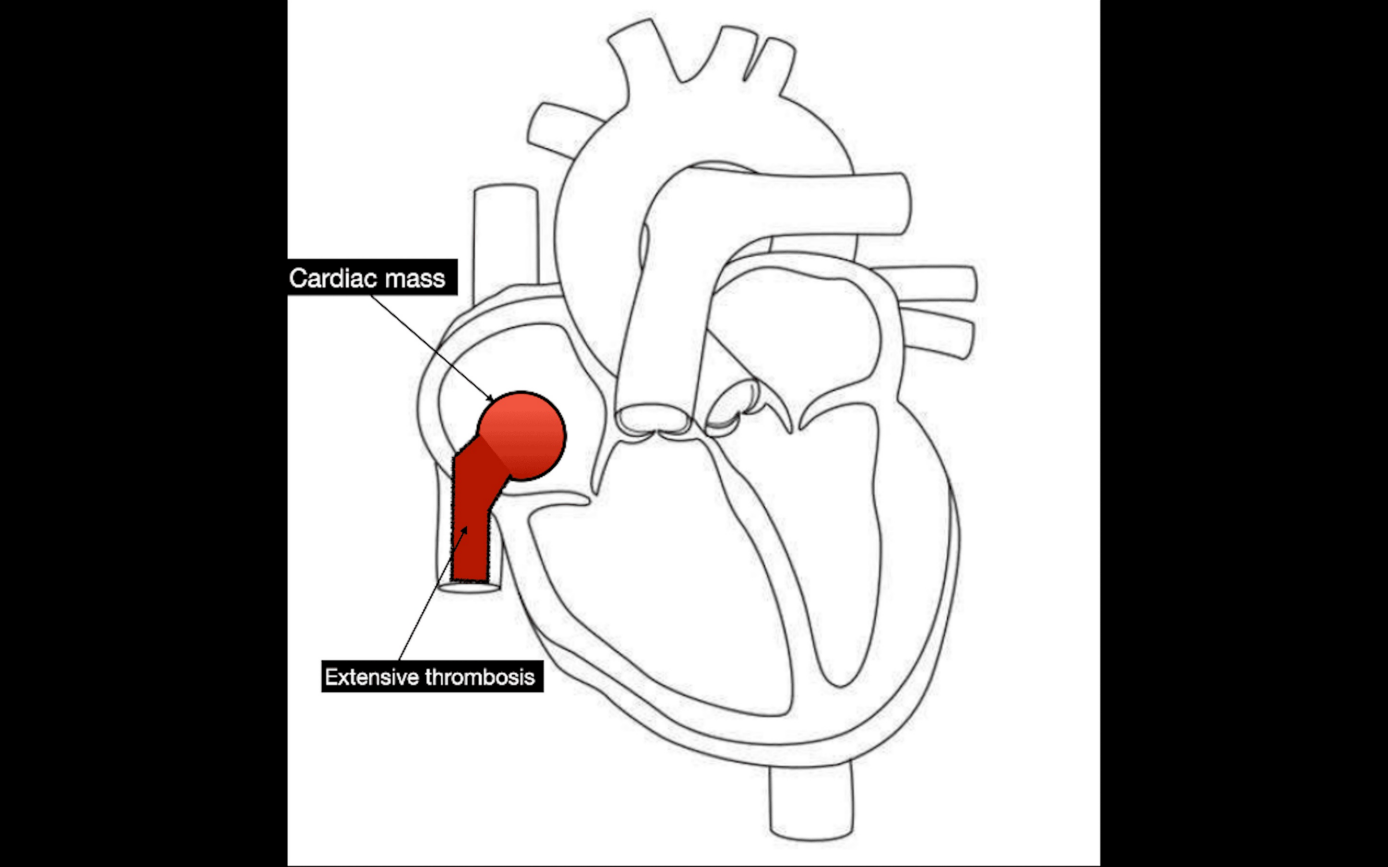
Diagrammatic representation of the right atrial tumor and its contiguous thrombotic extension into the inferior vena cava, illustrating the pathophysiology of Budd-Chiari syndrome. The diagram was created by the authors, adapted from template.net [9], a publicly available source.

A diagrammatic representation of the right atrial tumor with contiguous inferior vena cava (IVC) thrombosis, illustrating the pathophysiology of Budd-Chiari syndrome, is shown in Figure 1 [9].

Imaging findings

**Figure 2:**
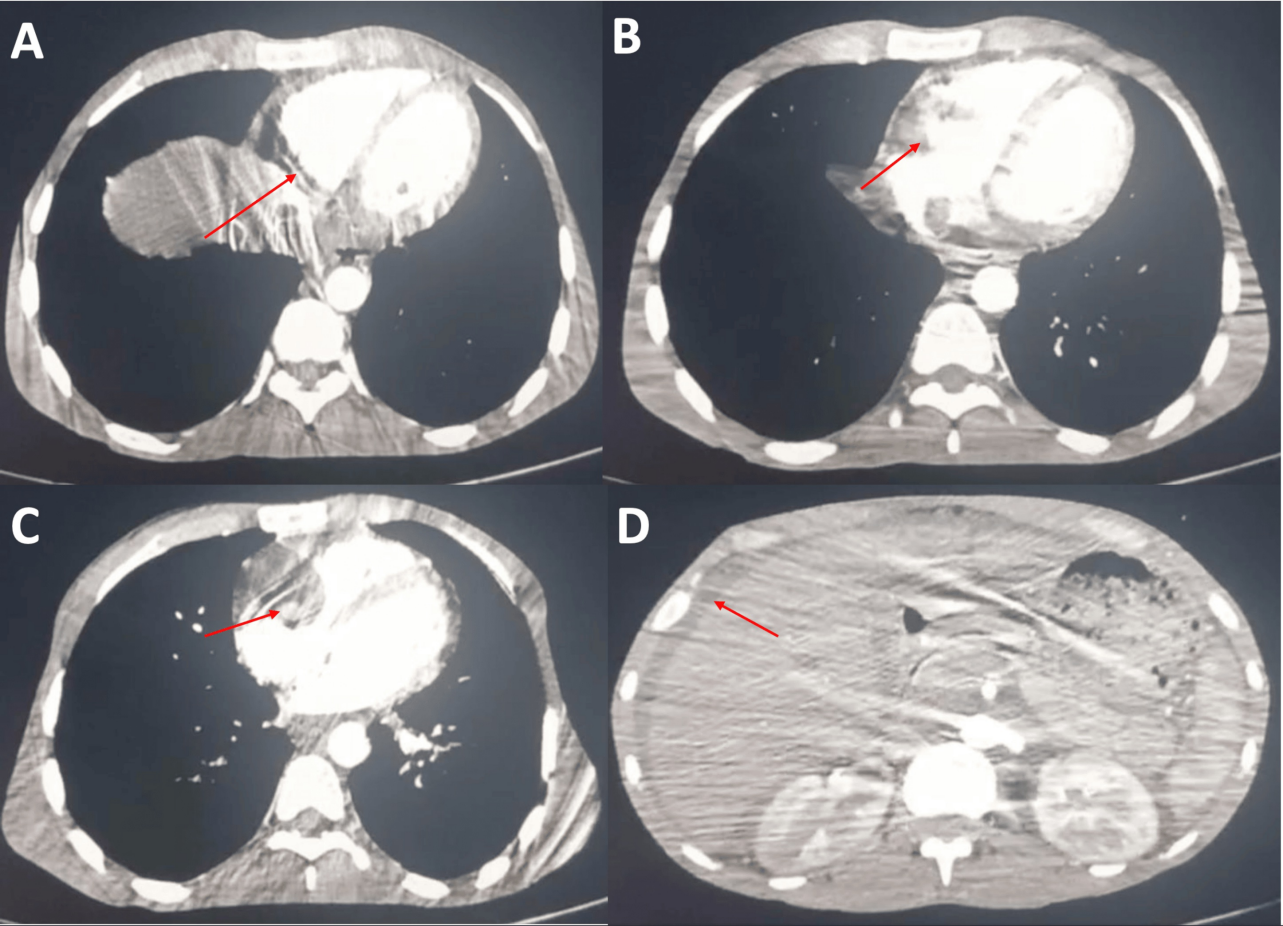
Abdominal CT: Extensive IVC thrombus with hepatic congestion and massive ascites. (A) Extensive thrombus within the supra-hepatic IVC (arrow). (B) Thrombus extending cranially toward the right atrium (arrow). (C) Irregular filling defect at the right atrium contiguous with the IVC thrombus(arrow). (D) Hepatic congestion with large-volume ascites. (arrow). Images were obtained from the authors’ institutional database. IVC, inferior vena cava

Abdominal CT (June 12, 2025) demonstrated *extensive thrombosis* of the IVC from its origin to below the hepatic veins, partial thrombosis of the left renal and iliac veins, hepatomegaly, and massive ascites consistent with Budd-Chiari syndrome. Importantly, no malignant lesions were detected in the digestive tract or pleuropulmonary regions, *excluding secondary cardiac involvement* (Figure 2).

**Figure 3:**
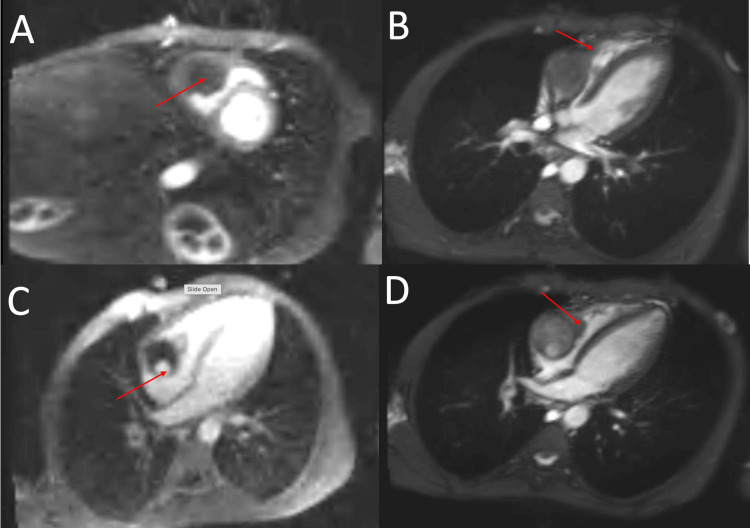
Cardiac MRI: Right atrial mass with features of malignant thrombus continuous with IVC thrombus. (A) Large atrial mass (arrow). (B) Partial prolapse into the right ventricle (arrow). (C) Heterogeneous right atrial mass with central hypointense thrombus and a small hyperintense viable component (arrow), prolapsing into the right ventricle. (D) Lobulated right atrial mass with irregular contours extending toward the tricuspid valve (arrow). Images were obtained from the authors’ institutional database. IVC, inferior vena cava

**Figure 4:**
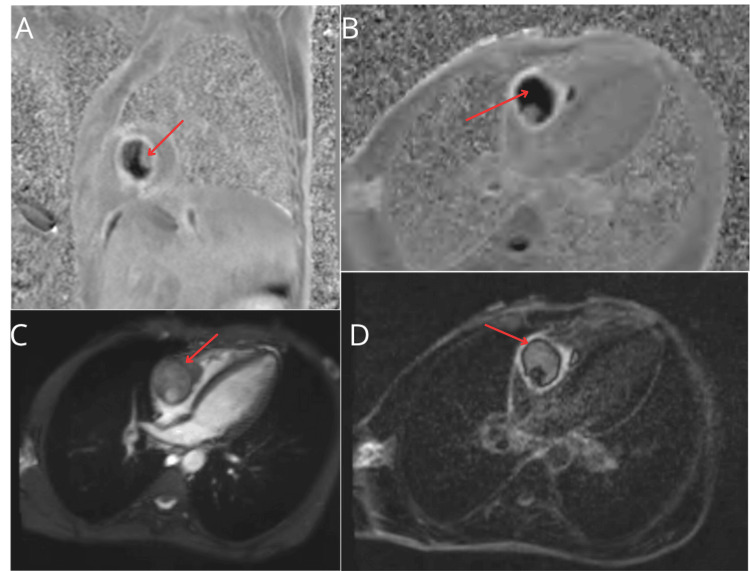
Cardiac MRI: (A-D) Right atrial mass with features of malignant thrombus continuous with IVC thrombus (arrows). Images were obtained from the authors’ institutional database. IVC, inferior vena cava

Cardiac magnetic resonance imaging (MRI) performed on June 26, 2025, revealed a *large, irregular right atrial mass* (62 × 41 mm) attached to the lateral wall at the base of the right ventricle. The mass partially prolapsed into the right ventricle and was continuous with the IVC thrombus, with surface ulceration and nodular enhancement suggesting malignant tumor thrombus. Right ventricular function was preserved (ejection fraction 58%). Subendocardial late gadolinium enhancement in the right coronary artery territory indicated ischemic injury, supporting the rationale for coronary angiography (Figures 3, 4).

**Figure 5:**
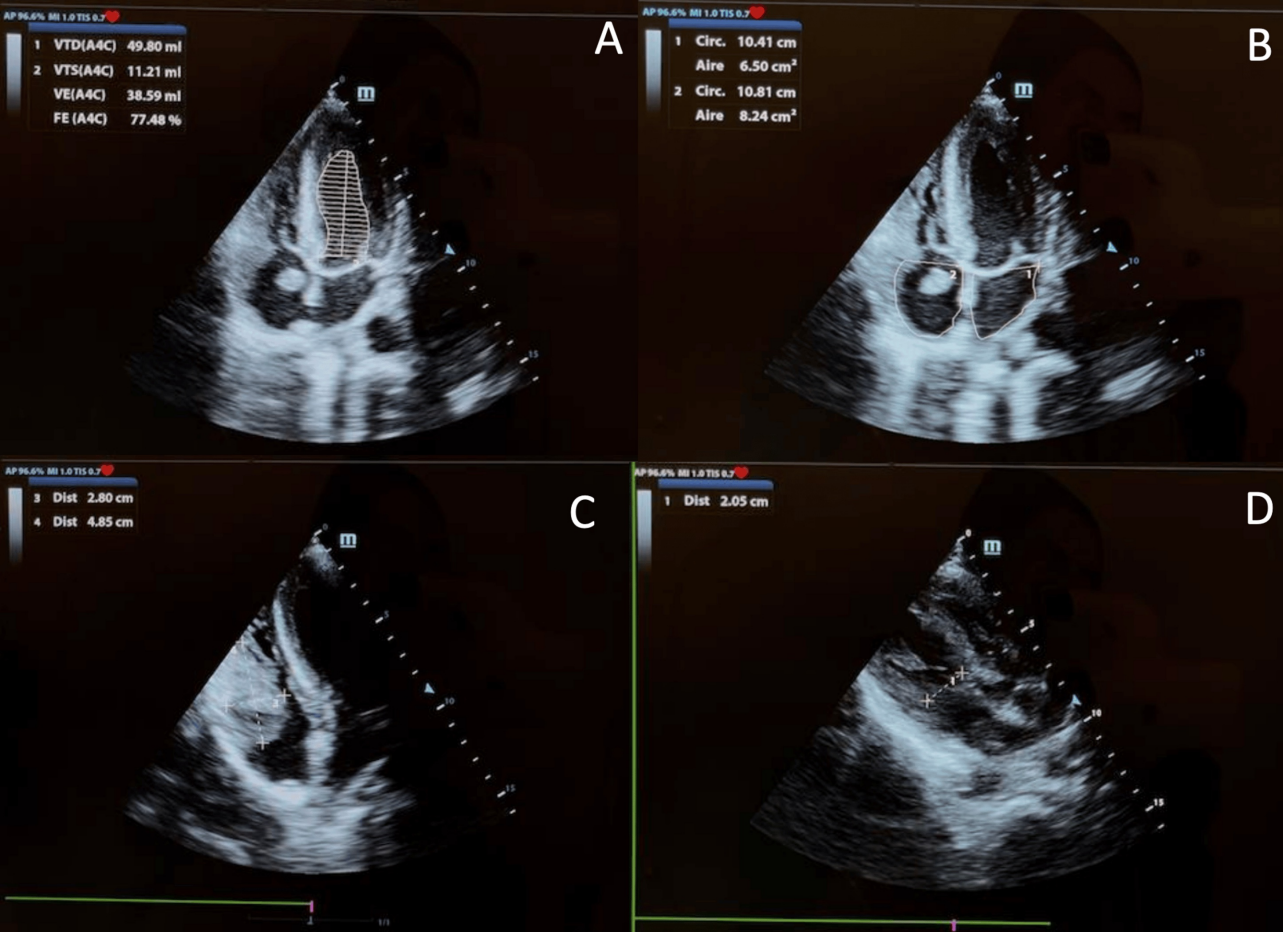
Echocardiography: RA and RV masses confirming MRI features; tumor thrombus suspected. (A) Preserved systolic function (LVEF 77%). (B) Normal atrial areas and a visible right atrial mass. (C) Extension into the right ventricle. (D) Preserved right ventricular size. Images were obtained from the authors’ institutional database. RA, right atrium; RV, right ventricle; MRI, magnetic resonance imaging; LVEF, left ventricular ejection fraction

**Figure 6:**
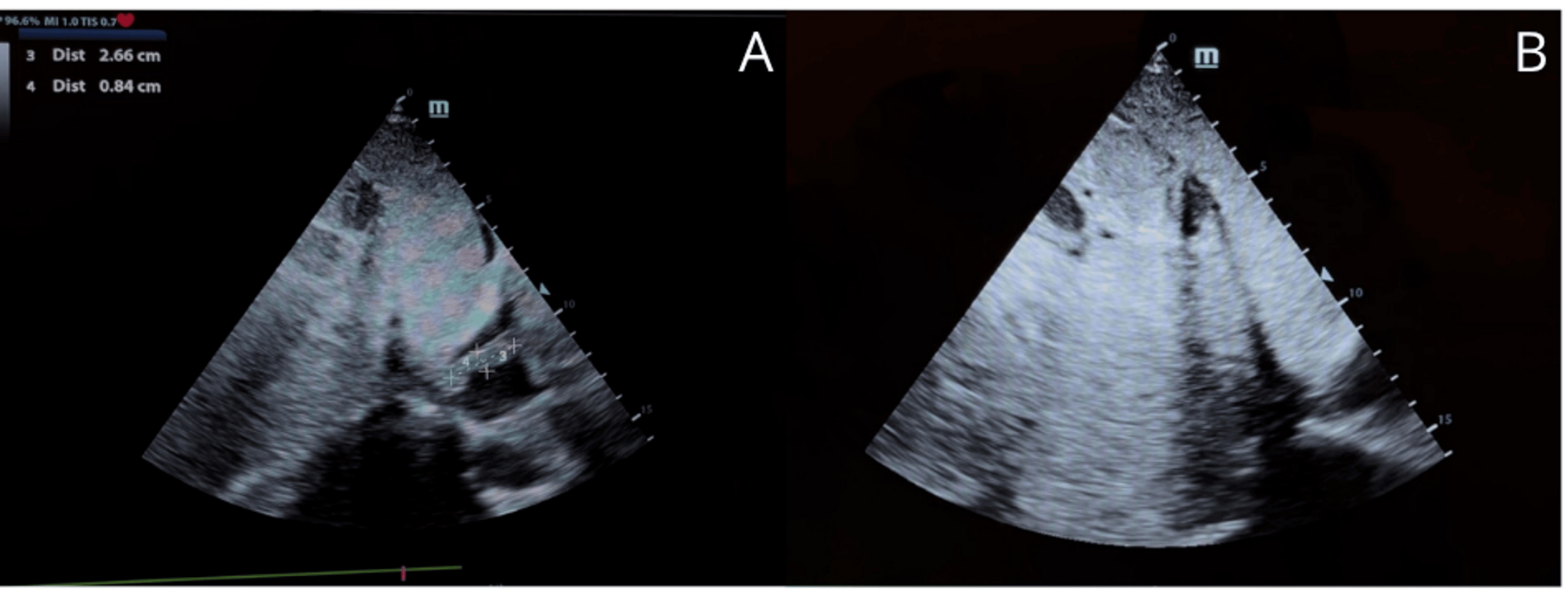
Echocardiography: RA and RV masses confirming MRI features; tumor thrombus suspected. (A) Right atrial mass extending without significant ventricular dilation. (B) Extension toward the inferior vena cava. Images were obtained from the authors’ institutional database. RA, right atrium; RV, right ventricle; MRI, magnetic resonance imaging

Transthoracic echocardiography confirmed right atrial and basal right ventricular masses with* irregular contours*, compatible with malignant tumor thrombus. Both left and right ventricular size and function were preserved, and no significant valvular abnormalities were identified (Figures 5, 6).

**Figure 7:**
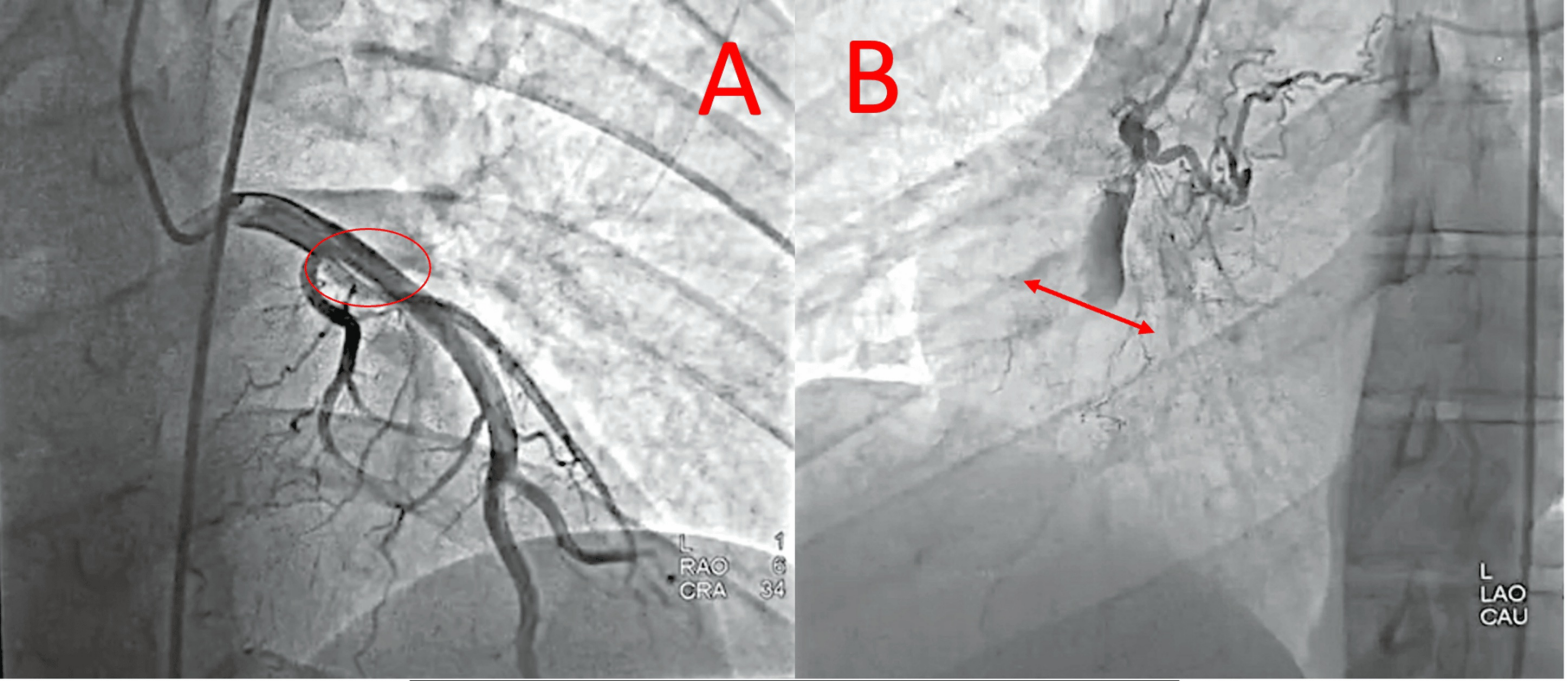
Coronary angiography: (A) Mid-LAD plaque (red circle), and (B) the chronic right coronary artery (RCA) occlusion (the red arrow), correlating with the ischemic MRI findings. Images were obtained from the authors’ institutional database. LAD, left anterior descending; RCA, right coronary artery; MRI, magnetic resonance imaging

**Figure 8:**
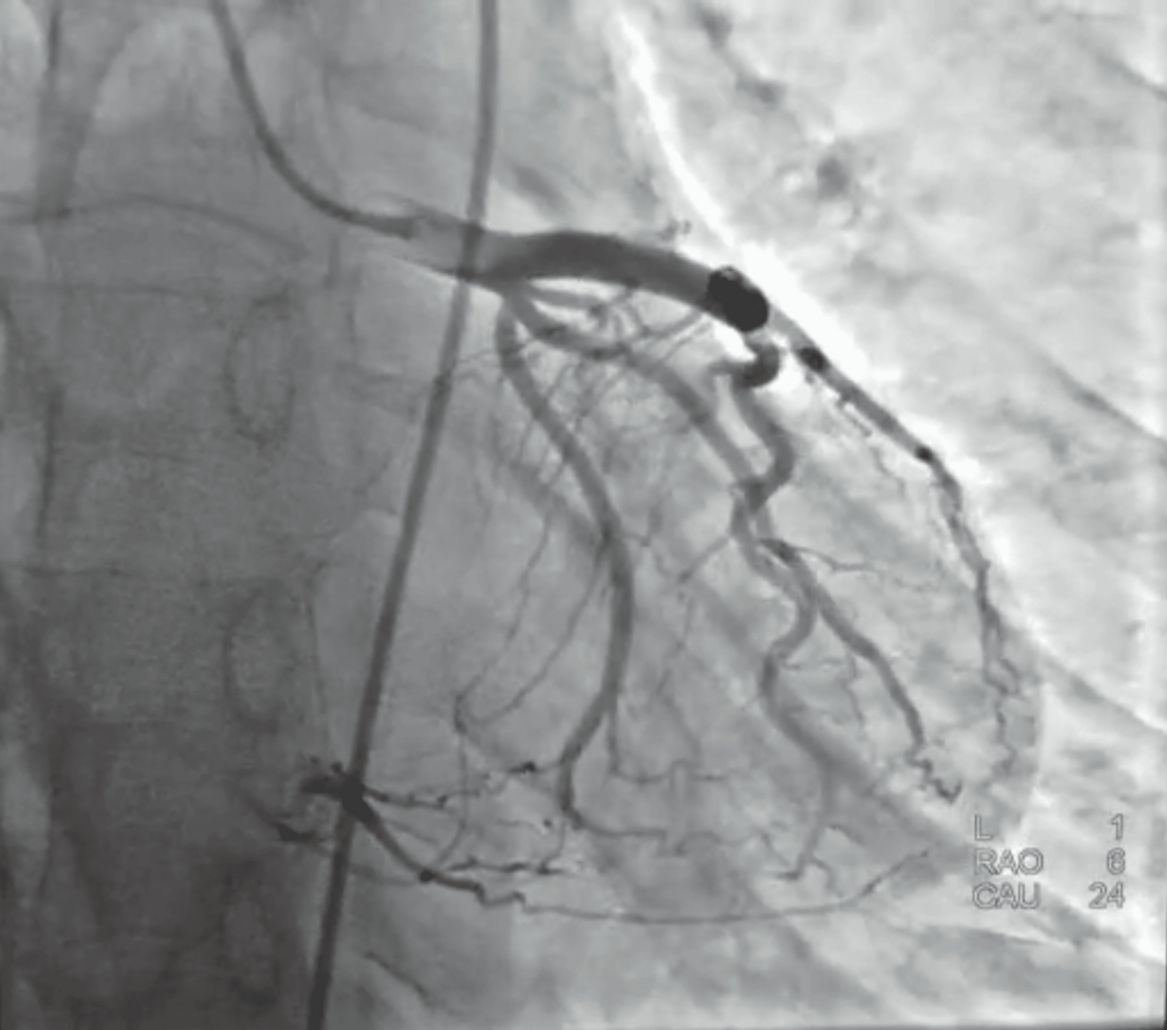
Coronary angiography: Mid-LAD plaque and chronic RCA occlusion correlating with ischemic MRI findings. Collateral circulation supplying the RCA territory from the LAD. Images were obtained from the authors’ institutional database. LAD, left anterior descending; RCA, right coronary artery; MRI, magnetic resonance imaging

Coronary angiography performed the same day showed a mid-left anterior descending (LAD) artery atheromatous plaque and chronic total occlusion of the proximal right coronary artery with collateral circulation, correlating with the ischemic changes observed on MRI (Figures 7, 8).

Clinical course

**Figure 9:**
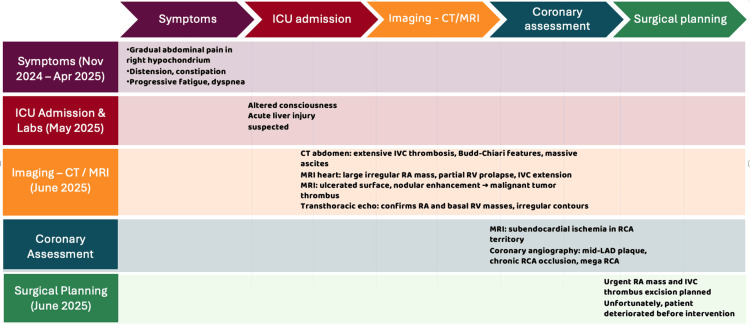
Timeline: Clinical presentation, labs, and imaging from symptom onset to ICU admission. Image credit: Timeline created by the authors from anonymized clinical data (original work). MRI, magnetic resonance imaging; ICU, intensive care unit

The patient was started on therapeutic anticoagulation. Urgent surgical excision of the right atrial mass and thrombus was planned due to malignant features and extensive IVC involvement. Unfortunately, the patient’s condition rapidly deteriorated, and he passed away before surgery could be performed. A timeline summarizing the clinical presentation, laboratory results, and imaging findings is shown in Figure 9.

## Discussion

Malignant cardiac tumors represent a very rare category of neoplasms and have a dismal prognosis, even with modern imaging and therapeutic progress [1,5]. Among these, cardiac angiosarcoma is the most frequent histological subtype. It usually originates from the right atrium and is often detected only at an advanced stage, largely because the early clinical manifestations are nonspecific or misleading [10,11].

Our case highlights the difficulty of diagnosing a tumor-related thrombus when it presents under the guise of Budd-Chiari syndrome. Although Budd-Chiari is most often caused by hepatic venous thrombosis, clinicians should also consider a cardiac malignancy, particularly in younger patients who present with widespread venous involvement and no evidence of primary hepatic or systemic malignancy [7,8].

Echocardiography, especially the transesophageal approach, remains the first-line modality for detecting intracardiac masses due to its high sensitivity [3]. Its ability to define tissue characteristics, however, is limited, which makes cardiac MRI indispensable. MRI can reveal heterogeneous T1/T2 signal patterns, areas of necrosis or hemorrhage, and late gadolinium uptake that are highly suggestive of angiosarcoma [12]. Contrast-enhanced CT is particularly useful for assessing extension into the vena cava and planning surgery [6], whereas PET/CT helps distinguish malignant from benign lesions and can uncover systemic metastases [13]. In our case, the MRI demonstrated nodular enhancement with continuity into the IVC thrombus-findings highly suggestive of a malignant cardiac mass and later consistent with the patient's clinical course, in the absence of any extracardiac primary malignancy. 

Review of literature indicates that right atrial tumors often manifest with features of systemic venous obstruction: Patel et al. described cases dominated by vena cava obstruction and pulmonary embolism rather than isolated cardiac complaints [14], Kim et al. reported a presentation with cardiac tamponade [6], and Yuan et al. observed rapid metastatic dissemination despite surgical resection [10].

In our case, the clinical presentation with hepatic venous outflow obstruction and severe ascites shared overlapping features with these reported cases, highlighting the diagnostic challenge and heterogeneity of initial manifestations.

Cardiac angiosarcoma originates from malignant transformation of vascular endothelial cells, characterized by aberrant angiogenesis, high mitotic activity, and a propensity for intravascular invasion [15]. Molecular studies revealed recurrent abnormalities such as p53 mutations, MDM2 amplification, and dysregulation of angiogenic pathways such as vascular endothelial growth factor (VEGF) signaling, which leads to aggressive progression and resistance to standard therapies [16]. These biological features explain the tendency for early metastasis (lungs, liver, bone) as well as the high rate of recurrence despite apparent complete surgical resection.

When large tumors occupy the right atrium, they can obstruct tricuspid inflow, cause functional tricuspid regurgitation, and raise systemic venous pressures. This hemodynamic impairment often results in hepatic congestion and ascites clinically mimicking manifestations of right-sided heart failure [8]. If the tumor extends near the conduction system, patients may develop arrhythmias or varying degrees of atrioventricular block, occasionally necessitating pacemaker implantation [17].

To this day, complete surgical resection with negative margins remains the only therapy shown to improve survival, with median overall survival ranging from 12 to 22 months in patients who undergo successful resection [16]. Unfortunately, the majority of patients are diagnosed at an advanced stage, when curative surgery is no longer possible [5].

Systemic chemotherapy with anthracyclines, ifosfamide, or taxanes may provide temporary disease control; however, objective response rates remain low, and treatment is often limited by cardiotoxicity [15].

More recently, emerging therapeutic approaches such as anlotinib (a multi-kinase angiogenesis inhibitor) and immunotherapy with PD-1/PD-L1 inhibitors have shown promise in refractory cases; however, published evidence remains limited to case reports and small-scale studies [16,18].

Despite multimodal therapy, the prognosis of these tumors remains poor. In fact, the majority of patients do not survive beyond one year [5,11].

In low- and middle-income countries, including North Africa, several barriers compound the poor prognosis of cardiac malignant masses.

In resource-limited settings, limited access to advanced imaging (particularly PET/CT), late presentation due to nonspecific symptoms and the limited availability of cardiothoracic oncology surgery all contribute to delayed diagnosis and restricted therapeutic options.

Promoting multidisciplinary collaboration and allowing access to modern diagnostic modalities represent essential steps toward improving management and survival of affected patients.

Study limitations

Our report is limited by the fact that it describes only a single patient, which prevents any generalization of outcomes. Moreover, histological confirmation wasn't obtained, as the patient died before programmed surgery could be performed. This lack of tissue diagnosis leaves some uncertainty; however, the clinical features and concordant multimodality imaging were highly suggestive of a malignant cardiac mass with canal extension. For these reasons, the findings of our study should be interpreted with caution.

## Conclusions

Malignant cardiac tumors carry an extremely poor prognosis, with most patients not surviving beyond one year despite multimodal therapy. This case highlights three important clinical lessons. First, cardiac malignancy should be suspected in young patients presenting with extensive venous thrombosis or Budd-Chiari syndrome. Second, the timely use of multimodality imaging is essential to distinguish tumor thrombus from bland thrombus and to guide management. Finally, coordinated multidisciplinary evaluation remains critical, even in situations where curative surgery is not feasible.
